# Chimeric Antigen Receptor-Modified T Cell Therapy in Metastatic Castrate-Resistant Prostate Cancer: Promise and Potential

**DOI:** 10.3390/cancers16051053

**Published:** 2024-03-05

**Authors:** Leah R. Tharian, Shiv Verma, Sanjay Gupta

**Affiliations:** 1 College of Arts and Sciences, Case Western Reserve University, Cleveland, OH 44016, USA; lrt42@case.edu; 2 Department of Urology, School of Medicine, Case Western Reserve University, Cleveland, OH 44106, USA; sxv304@case.edu; 3 The Urology Institute, University Hospitals Cleveland Medical Center, Cleveland, OH 44106, USA; 4 Department of Pharmacology, Case Western Reserve University, Cleveland, OH 44016, USA; 5 Department of Pathology, Case Western Reserve University, Cleveland, OH 44016, USA; 6 Department of Nutrition, Case Western Reserve University, Cleveland, OH 44016, USA; 7 Division of General Medical Sciences, Case Comprehensive Cancer Center, Cleveland, OH 44106, USA

## 1. Introduction

Prostate cancer, the most common cancer among males, has a mortality rate of approximately 29,000 deaths each year in the United States alone [1]. While localized cases can be treated with surgery or radiation therapy, roughly 30% of patients will develop metastatic disease. For these patients, the most common treatment is androgen deprivation therapy (ADT). However, many of these patients relapse, unavoidably progressing to metastatic castration-resistant prostate cancer (mCRPC). The time to progression from hormone-sensitive prostate cancer to CRPC is about 16.5 months [2]. The prognosis of patients with mCRPC is poor, with a median survival of 10 to 21.7 months and a survival rate of 30% over 5 years [3]. The introduction of novel anti-androgen agents and androgen synthesis inhibitors has significantly increased the progression-free survival (PFS) and overall survival (OS) of patients with mCRPC. However, ~20–40% of patients develop primary resistance to these agents, and nearly all patients will progress to secondary resistance [4]. Other therapies approved by the Food and Drug Administration for the treatment of mCRPC include radiopharmaceutical agents that allow for targeted radiotherapy to metastatic sites. Among these are Radium-233, vaccine-based therapy such as Sipuleucel-T, Poly(ADP-ribose) polymerase (PARP) inhibitors, and immune checkpoint inhibitors. While these treatments can increase overall survival rates, they are not a perpetual cure for the disease, and have been shown to only have temporary benefits with significant adverse effects [5]. It is this lack of effectiveness of the agents currently in use, as well as the promise shown by adoptive immunotherapy, such as chimeric antigen receptor (CAR)-modified T cells, in the treatment of hematological malignancies [6,7,8], that have brought focus to the use of CAR-T cells in the treatment of solid tumors such as mCRPC.

A phase I study conducted by Narayan et al. [9] presented the results of a trial of CRPC-targeting CAR-T cells directed against prostate-specific membrane antigen (PSMA), thus addressing the challenges of the highly immunosuppressive tumor microenvironment (TME) with a dominant negative form of transforming growth factor-β (TGF-β)RII engineered CAR-T cells [10]. The authors engineered CAR-T cells that were directed against PSMA, which is expressed in prominent levels in mCRPC and has shown promising signs as a tumor-associated antigen (TAA) for immunotherapy [11,12,13,14,15,16]. The study is unique in that it is the first clinical trial involving the use of CAR-T cells optimally engineered with a dominant negative TGFβR (TGFβRDN) in an attempt to neutralize the components of the TME in mCRPC patients. It has been reported that TGFβRDN supports in vivo proliferation and memory differentiation, decreases exhaustion, and promotes the induction of tumor eradication [17,18]. 

## 2. Background

CAR-T cell therapy involves the formation of autologous, genetically engineered T cells that attack specific tumor-associated antigens (TAAs) [7]. CAR-T cells are generated via leukapheresis of the patient’s blood, from which T cells are isolated and then transfected using a viral or non-viral vector with the genetically engineered CAR protein. After transfection, T cells are subjected to molecular expansion and ex vivo purification. Finally, the CAR protein-expressing T cells (known as CAR-T cells) are then readministered to the patient. The structure of a CAR-T cell consists of three main segments and includes an extracellular domain, a transmembrane domain, and an intracellular zone. The extracellular domain consists of a single-chain fragment variable (scFV) that binds to a TAA. The transmembrane domain, which links the scFV and the T cell, is made up of proteins (such as FcεRI, CD3, CD8, and CD28) that are attached to an intracellular domain. The intracellular zone harbors the immune receptor tyrosine-based activation motif (ITAM), which plays a significant role in signal transduction for T cell activation. In initial in vivo tests, CAR-T cell structure displayed poor T cell activation and persistence, which led to the addition of several molecules to the intracellular domains upon further development. The latest fourth-generation CAR-T cells, called “TRUCKs” (T cells redirected for universal cytokine mediated killing), contain many motifs within the intracellular cassette which help combat the immunosuppressive tumor microenvironment, resulting in enhanced therapeutic effectiveness as well as preventing excessive toxicity [7,8,9,10].

The authors conducted a phase I clinical trial that was designed to study the efficacy, safety, and dosage of PSMA-TGFβRDN autologous CAR-T cells administered to patients with and without lymphodepletion [9]. The study was executed through a 3 + 3 dose escalation module. Of the eighteen patients enrolled in the trial, five failed initial screenings. The remaining thirteen patients were then divided into three cohorts. On day 0, cohort 1 received a single dose of 1–3 × 10^7^/m^2^ cells without lymphodepleting chemotherapy (LD), and cohort 2 subjects received a single dose of 1–3 × 10^8^/m^2^ cells without LD. All cohort 3 patients underwent LD with cyclophosphamide and fludarabine and in addition, received infusion of either 1–3 × 10^7^/m^2^ or 1–3 × 10^8^/m^2^ cells. The median age of participants upon entry was 70 years (interquartile range (IQR), 57–72 years), and the median serum PSA level was 36.6 ng/mL. Eight patients (61.5%) presented with stage 4 disease at initial diagnosis, thirteen (100%) had previously received androgen receptor signaling inhibitor therapy, and six (46.2%) had previously received docetaxel chemotherapy. The median number of previous therapies for CRPC was two, with a range of one to eight previous treatment lines. These patients were then followed up in the short term with computed tomography of the chest/abdomen/pelvis, bone scans, and PSA levels every 3 months for a period of 2 years. Then, long-term follow-up was maintained for up to 15 years. In cohort 1, no treatment-related grade ≥3 adverse effects (AEs) or cytokine release syndrome (CRS) were observed. In cohort 2, two subjects developed grade 3 CRS, with one of them also manifesting immune effector cell neurotoxicity syndrome. However, these AEs fully resolved within twenty-four hours following tocilizumab and corticosteroid therapy. In cohort 3, where all subjects were lymphodepleted, one subject who received the higher dose of the infusion therapy died. Subsequently, additional three subjects were treated under an amended protocol at a modified, deescalated dose of 1–3 × 10^7^/m^2^, with no grade ≥3 AEs observed. A 30% decline in PSA levels, which was used as a marker of anti-tumor response, was observed in 30% of infused subjects with a higher frequency of PSA decline in dose-level cohorts with LD. In total, at the 3-month imaging follow-up, 38.5% of the subjects were in the “stable disease” category of the Prostate Cancer Working Group’s criteria for optimal radiographic response assessment. Median overall survival was 477 days (15.9 months), and progression-free survival was 132 days (4.4 months) in the 13 subjects.

## 3. Discussion

The study showed that infused CAR-T cells were tolerated and generally safe at doses below the threshold of the dose-limiting toxicity level of 1–3 × 10^8^/m^2^. On analysis of the post-infusion kinetics of peripheral CAR-T PSMA-TGFβRDN cell expansion, it was observed that there was an initial expansion of the peripheral armored CAR-T cell line, which peaked within the first 14 days post infusion, followed by a subsequent decline in cell levels over the following days. It was also observed that there was a direct correlation between CAR-T PSMA-TGFβRDN cell expansion and higher administered doses. LD was also noted to be a factor in achieving higher levels of CART-PSMA-TGFβRDN cell expansion. These findings are indicative of the successful use of TGFβRDN as a functional “armor” in the CAR construct to attenuate a common immunosuppressive barrier in the TME of mCRPC with the goal of potentiating improved proliferation, effector cytokine elaboration, and CAR-T cell persistence.

A major strength of CAR-T cell therapy is that specific tumor antigens can direct an immunological response without antigen-presenting cells (APCs) or the major histocompatibility complex system (MHC) [19]. Since the MHC system is not required for antigen presentation and processing, CAR-T cells are insensitive to tumor escape mechanisms commonly facilitated by the MHC. These mechanisms recognized in prostate cancer include the downregulation of MHC Class I expression or the manipulation of the TME so that there is fewer tumor–MHC Class I epitope interactions [20]. In addition, CARs can target antigens such as glycolipids, glycosylated proteins, and conformational epitopes that are not readily recognized by T cell receptors. Therefore, the administration of CAR-T cells to patients both bypasses immunological tolerance and enhances tumor antigen targeting [21].

There are few limitations in the study, including its small sample size and the inclusion of subjects with extensive prior treatment histories. Although the study demonstrated the feasibility and safety of PSMA-redirected TGF-β-resistant CAR-T cells in the treatment of mCRPC as well as the importance of LD in the enhancement of CAR-T cell proliferation and function, there still exist some confines, including (i) off-target effects such as cytokine release syndrome, which has the potential for causing fatal outcomes, (ii) antigen “escape” due to higher instances of antigenic heterogeneity in solid tumors compared to hematological malignancies, (iii) off-tumor/on-target effects due to the presence of TAA in normal tissues, (iv) poor trafficking/tumor infiltration of CAR-T cells, and (v) insufficiency of T cell recruitment to the tumor site due to immunosuppressive tumor microenvironment.

## 4. Conclusions and Future Prospects

Prostate cancer patients have not seen benefits from the use of immunotherapy such as checkpoint inhibitors or sipuleucel-T where no decrease in PSA or radiographic responses are recorded. While the results of Narayan et al. [9] are encouraging, there is need for additional studies in order to determine the most efficacious treatment combinations, reduce side effects, and develop an individualized therapeutic regimen for this deadly form of prostate cancer.
